# Native Valve Aspergillus Endocarditis in a Non-Neutropenic Immunocompromised Patient on Anti-TNF α Blockers Therapy

**DOI:** 10.7759/cureus.20629

**Published:** 2021-12-23

**Authors:** Joana Azevedo Carvalho, Leonor Boavida, Isabel Amorim Ferreira, Bruno Grima, José Delgado Alves

**Affiliations:** 1 Department of Medicine IV, Hospital Professor Doutor Fernando Fonseca, Amadora, PRT; 2 Intensive Care Unit, Hospital Professor Doutor Fernando Fonseca, Amadora, PRT; 3 Systemic Immune-Mediated Diseases Unit (UDIMS) Department of Internal Medicine IV, Hospital Professor Doutor Fernando Fonseca, Amadora, PRT

**Keywords:** anti-tnf agents, autoimmune diseases, seronegative spondyloarthropathy, fungal endocarditis, invasive aspergillosis

## Abstract

Invasive aspergillosis is a rare opportunistic infection mainly occurring in patients with a well-established risk such as neutropenia or conditions that lead to chronically impaired cellular immune responses. Systemic corticosteroids are a well-known risk factor for fungal infections. Recently, reports of invasive aspergillosis in patients treated with monoclonal biologic agents, such as tumor necrosis factor-alpha inhibitors, have been increasing.

We present the case of a 47-year-old female patient with seronegative spondyloarthropathy treated with infliximab and corticosteroids. The patient presented classical symptoms of an acute lower respiratory infection, and she was treated with a β-lactam antibiotic. Infliximab administration was deferred until nine days after clinical recovery. Fourteen days after drug administration, she was admitted with a symptomatic subcortical hematoma in the left parietal region. There was a rapid neurological recovery, and there were no risk factors for haemorrhagic stroke detected. The chest X-ray revealed an oval mass with an air crescent sign, and the CT scan was suggestive of aspergilloma. Bronchoalveolar lavage cytology identified *Aspergillus* spp. Voriconazole was initiated and, after one month of treatment, the patient was readmitted with a left facial palsy associated with hemiparesis and dysarthria. Laboratory evaluation showed leukocytosis and elevated C-reactive protein. A severe right middle cerebral artery stroke was present on the brain CT scan. Transesophageal echocardiogram revealed large mitral valve vegetation, and the diagnosis of *Aspergillus* endocarditis with cerebral embolization was made.

Fungal infections are challenging due to the diagnosis infrequency and paucisymptomatic natural history. Despite being crucial in the treatment of autoimmune diseases, immunosuppressive drugs increase the risk of fungal infection. It is extremely important to consider *Aspergillus* infection in immunosuppressed patients, and the need for prophylaxis in non-neutropenic patients with risk factors should be clarified.

## Introduction

*Aspergillus conidia* are ubiquitous in the soil, and the vast majority of the population is exposed, but it does not result in colonization. For persons who do acquire and retain conidia, a spectrum of clinically significant outcomes can occur, from asymptomatic colonization to invasive infection. Dissemination to multiple organ systems occurs in up to 10% of patients by haematogenous spread [1,2].

Recognized populations at risk of invasive *aspergillosis* (IA) are patients with haematological malignancy, solid organ transplantation recipients, patients treated with prolonged high dose corticosteroids or with other immunosuppressants, patients with advanced AIDS or neoplasia, chronic obstructive pulmonary disease, liver failure, liver cirrhosis, influenza and critically ill patients requiring admission to intensive care [3]. Fungal endocarditis is a rare disease with a poor prognosis. It corresponds to 1%-10% of all infective endocarditis and is associated with high mortality (>50%) and morbidity. *Aspergillus spp.* accounts for 20%-25% of the cases [4].

We present a case of an IA in an immunosuppressed non-neutropenic patient manifested as native valve endocarditis with systemic embolization. This case was previously presented as an e-poster at the 18^th^ European Congress of Internal Medicine on August 30, 2019.

## Case presentation

A 47-year-old woman with a seronegative spondyloarthritis treated with prednisolone (7.5mg/day) and infliximab (5mg/Kg every 8w) presented to the walk-in clinic of the systemic autoimmune disease unit with low-grade fever, productive cough, and wheezing. The X-ray revealed a well-circumscribed parenchymal opacity in the left lung. The diagnosis of bacterial pneumonia was made, and she was empirically treated with amoxicillin-clavulanic acid. Infliximab administration was deferred until nine days after her clinical recovery, and the re-administration occurred without any immediate complication.

**Figure 1:**
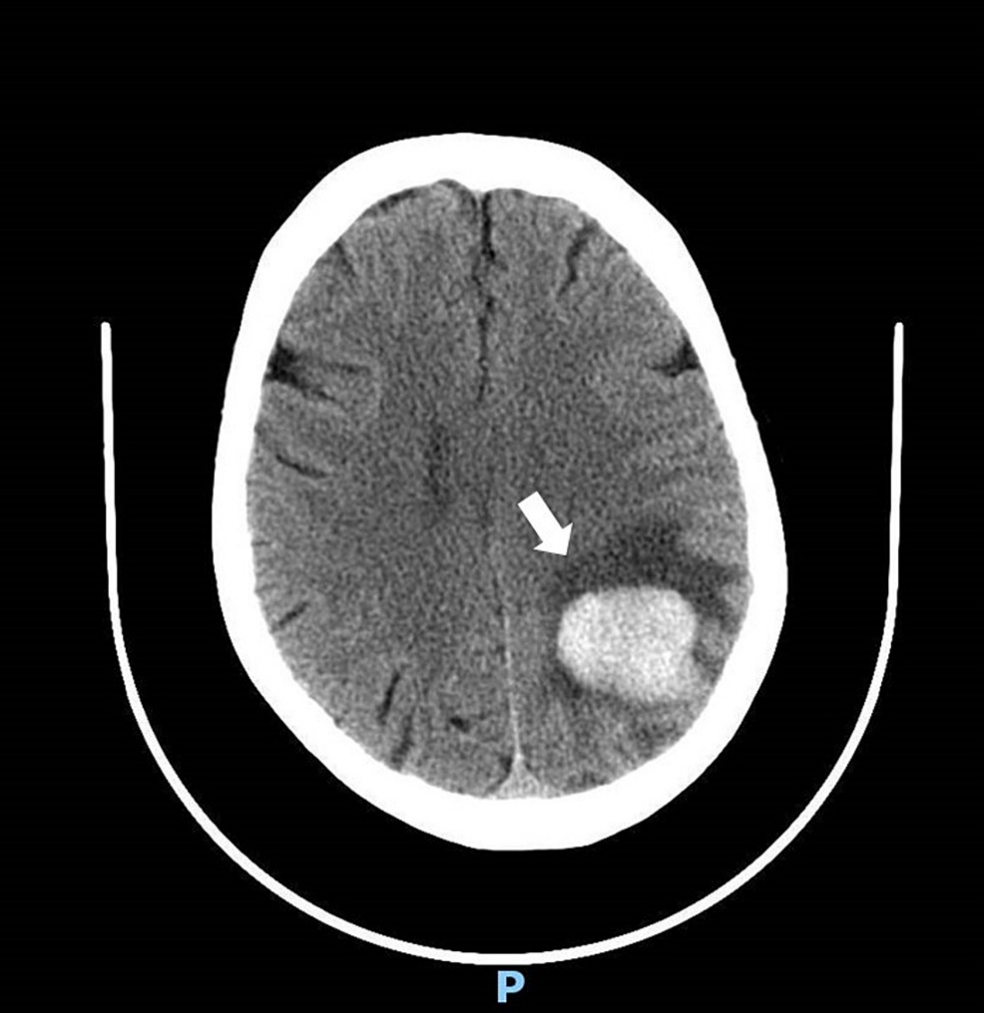
Cranial CT scan showing an acute intracerebral haemorrhage with vasogenic edema

Fourteen days after drug administration, she was admitted with a frontal pulsatile headache, dysarthria, and cognitive impairment that lasted for 24-hours. On clinical examination, she was normotensive and had impaired attention, anomia aphasia, agraphia, alexia, and left homonymous hemianopia. The brain CT scan showed a cortico-subcortical hematoma in the left parietal region with vasogenic edema (Figure 1).

**Figure 2:**
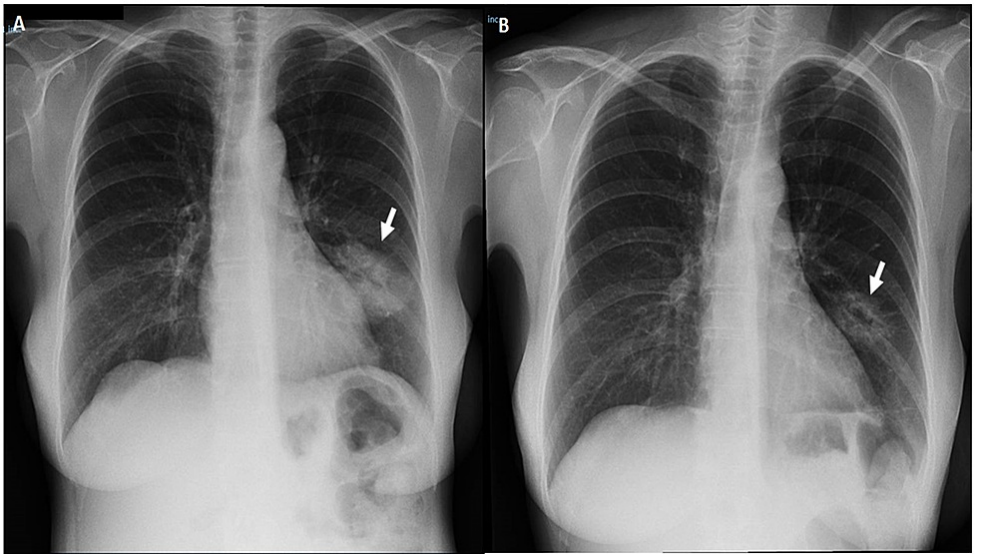
X-ray showing typical plain radiographic findings of aspergilloma A: The first X-ray assumed as a bacterial pneumonia showing a intrapulmonary soft tissue-like mass B: X-ray after 23 days showing a crescentic lucency surrounding the peripheral aspect of the mass

The patient had mild hypertension, controlled with ramipril 5mg once daily (od) and bisoprolol 5mg od. Arteriovenous malformations were excluded by CT angiography. Although no respiratory symptoms had been reported then, the chest X-ray showed an oval mass with an air crescent sign in the left lung (Figure 2).

**Figure 3:**
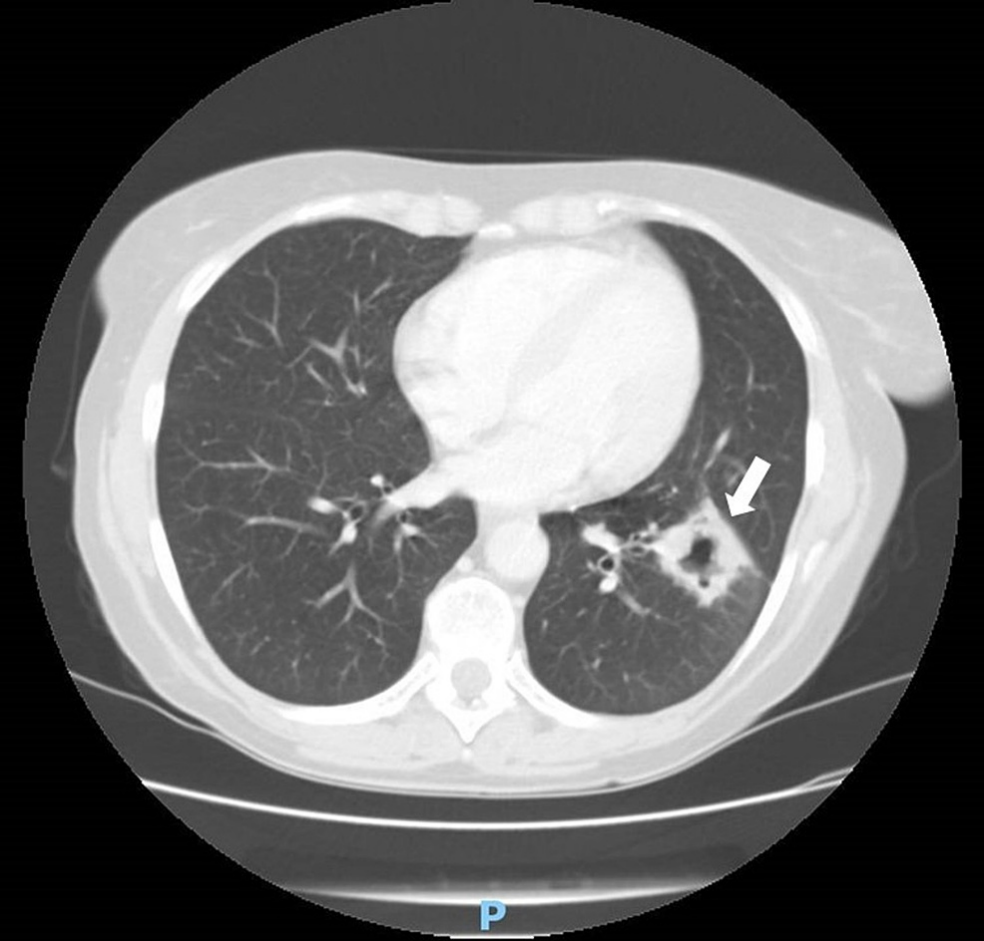
Thoracic CT scan showing rounded mass within a cavity, typical of aspergilloma (arrow)

The thoracic CT scan revealed typical radiographic findings of aspergilloma (Figure 3). 

*Aspergillus spp.* was identified on bronchoalveolar lavage cytology, and treatment with voriconazole was started. There was a complete recovery of the neurological symptoms, and the patient was discharged from the hospital. During the follow-up period, the patient had left panuveitis treated with the combined treatment of topical corticosteroids, and the oral prednisolone dose was increased to 10mg.

After one month of treatment, the patient was readmitted with left facial palsy, homolateral hemiparesis, and dysarthria started upon waking up. On neurological examination, she presented somnolence, dysarthria, left central facial palsy, left hemiplegia associated with hemihypesthesia, a left positive Babinski sign, and myoclonic seizures. The suspicion of a Wake-Up stroke was confirmed with a severe right middle cerebral artery stroke (ASPECT 3) on the CT scan.

**Figure 4:**
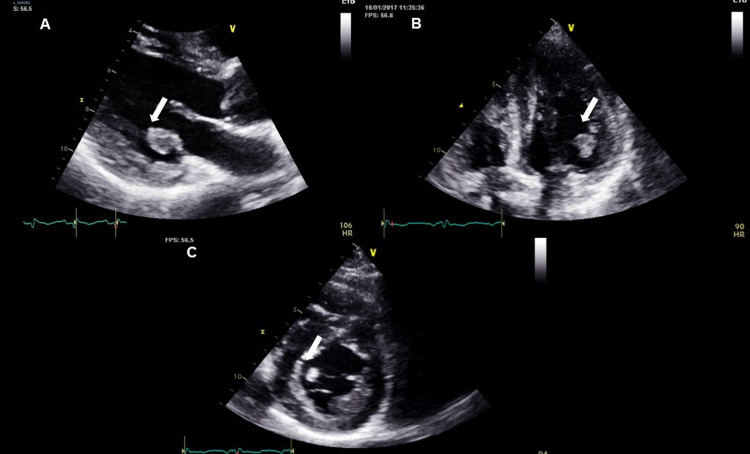
Aspergillus vegetation (arrow) of native mitral valve on echocardiogram A: Parasternal long axis view, B: Apical 4-chamber view, C: Parasternal short axis view

Laboratory investigation showed a white blood cell count of 23,000cells/mm3 (84.5% neutrophils, 1.1% eosinophils and 8.5% lymphocytes) associated with elevation of C-reactive protein and erythrocyte sedimentation rate (21mg/dl and 92mm/h, respectively). No fever was reported. However, physical examination revealed an abnormal systolic heart murmur, Janeway lesions, and Osler’s nodes. Roth spots were not found on the fundoscopic exam. Blood cultures were negative for bacterial or fungus, and serum galactomannan antigen was also negative. The transoesophageal echocardiogram showed a large and highly mobile vegetation on the mitral valve (11x13mm) (Figure 4).

The diagnosis of fungal endocarditis with cerebral embolization was made. Broad-spectrum antibiotic therapy with meropenem and vancomycin was introduced in association with voriconazole and caspofungin due to the risk of bacterial superinfection in an immunosuppressed patient. Prophylactic treatment with levetiracetam was started due to the potential epileptogenic ischemic lesion. The large ischemic stroke (ASPECT 3) was pointed as a major contraindication for cardiothoracic surgery due to the haemorrhagic risk during the extracorporeal circulation.

No clinical or laboratory responses were seen. After 16 days of admission, vegetation embolization occurred, causing a catastrophic right ischemic stroke. The patient was admitted to the ICU because of the necessity of vasopressor, neurological, and ventilatory support. She was discharged after 14 days with major neurological impairment and died six weeks later in a nursing home.

## Discussion

Acute IA is a devastating opportunistic infection, and its diagnosis can be challenging and laborious. IA presents most frequently with respiratory symptoms, such as fever, cough, chest or pleuritic pain, shortness of breath, and/or haemoptysis. A paucisymptomatic presentation is common in immunosuppressed or neutropenic patients due to the lack of inflammatory response, precluding early diagnosis [1,2]. The severity of the invasive infection correlates inversely with the immune status of the host. *Aspergillus* endocarditis is one of the more severe forms of *Aspergillus* infection [5,6].

Despite having a similar clinical presentation as bacterial endocarditis, fungal endocarditis is more commonly associated with neurological complications, such as bulky embolization, ischemic or haemorrhagic stroke, and mycotic aneurysm [4].

In this case, despite the adequate treatment of pulmonary aspergillosis with voriconazole, the patient evolved with *Aspergillus* endocarditis with major neurological complications. Drug-induced immunosuppression was the major risk factor. Immunomodulators and immunosuppressants used for the treatment of autoimmune diseases, in particular corticosteroids, have been associated with cases of aspergillosis. An incidence of IA of 6.19 cases to 8.63 cases/ 100,000 persons have been described in patients treated with tumor necrosis factor α-blockers (i.e., infliximab, adalimumab, etanercept, golimumab, and certolizumab pegol) [2].

Imaging is a critical component in diagnostic evaluation. Typical thoracic CT findings pointed to aspergilloma, and a positive microscopic examination confirmed the diagnosis in our patient. Classical CT findings of IA include macronodule(s) >1 cm, which may be surrounded by a halo of ground-glass attenuation (halo sign), pleural based wedge-shaped areas of consolidation, alveolar consolidations, masses (especially in solid organ transplant recipients), internal low attenuation, reverse halo sign, cavity or air-crescent sign (delayed finding), ground-glass opacities and pleural effusion [3].

Blood cultures have a low sensitivity for *Aspergillus* infection (estimated to be only 4%). Galactomannan is an important constituent of the cell wall of *Aspergillus*. Positive galactomannan detection in fluids (especially in bronchoalveolar lavage) is considered more sensitive than cultures for the diagnosis of IA. The sensitivity of the test could be influenced by the immune status of the host, the site of involvement, prior antifungal prophylaxis or treatment, the sample type, and the experience of the laboratory performing the test. In this case, the serum galactomannan was negative, and this could be explained by a sensitivity reduction after antifungal exposure. Demonstration of tissue invasion by hyphae through microscopic examination of biopsy provides a diagnosis of proven invasive fungal infection. Molecular detection of fungi on hyphal positive biopsy samples is strongly recommended, although not available in many centres [1,3,4].

Voriconazole monotherapy is the first-option antifungal therapy in non-haematological patients, and it has been associated with reduced mortality. For patients with IA refractory or intolerant to voriconazole, a lipid formulation of amphotericin B or an echinocandin can be used. Caspofungin has been approved for salvage therapy in IA, but efficacy rates are only around 33%. Combination therapy with voriconazole and an echinocandin may provide a benefit over monotherapy in some patients. However, the evidence supporting its use is controversial and additional studies are needed to further explore the benefit of combination therapy [1,2,5,7].

Infection-related mortality remains globally high. Delayed diagnosis of IA in non-neutropenic patients is associated with a bad prognosis, with mortality rates exceeding 80%-90% [6].

## Conclusions

There are several challenges in the diagnosis and management of IA. IA incidence is increasing due to an emerging “population at risk” that includes patients with a severe chronic obstructive pulmonary disease requiring high-dose steroid therapy, Child-Pugh C hepatic cirrhosis, patients in intensive care units, and systemic diseases requiring immunosuppressive therapy. Fungal infections complicating therapy with Infliximab have been reported sporadically, but systemic corticosteroids are an undeniable risk factor. Nevertheless, an early diagnosis may not be easy to confirm, and despite the best treatment regimens available, severe morbidity and mortality are still exceedingly high. This being the case raises the concern and debate about whether anti-fungal prophylaxis should be considered in such very-high risk patients.
